# Single Nucleotide Polymorphism Typing of *Bacillus anthracis* from Sverdlovsk Tissue

**DOI:** 10.3201/eid1404.070984

**Published:** 2008-04

**Authors:** Richard T. Okinaka, Melinda Henrie, Karen K. Hill, KristinS. Lowery, Matthew Van Ert, Talima Pearson, James Schupp, Leo Kenefic, Jodi Beaudry, Steven A. Hofstadler, Paul J. Jackson, Paul Keim

**Affiliations:** * Los Alamos National Laboratory, Los Alamos, New Mexico, USA; † Northern Arizona University, Flagstaff, Arizona, USA; ‡ Ibis Biosciences Inc, Carlsbad, California, USA; § Lawrence Livermore National Laboratory, Livermore, California, USA; ¶ Translational Genomics Research Institute, Phoenix, Arizona, USA

**Keywords:** Sverdlovsk, anthrax, *Bacillus anthracis*, canSNP, *pagA*, dispatch

## Abstract

A small number of conserved canonical single nucleotide polymorphisms (canSNP) that define major phylogenetic branches for *Bacillus anthracis* were used to place a Sverdlovsk patient’s *B. anthracis* genotype into 1 of 12 subgroups. Reconstruction of the *pagA* gene also showed a unique SNP that defines a new lineage for *B. anthracis*.

The 1979 accidental release of *Bacillus anthracis* spores in the northeastern quadrant of Sverdlovsk (today’s Ekaterinburg) in the former Soviet Union resulted in a relatively large-scale incident of inhalation anthrax (*1*). Histologic examinations of formalin-fixed, paraffin-embedded tissue samples from patients affected by this incident showed pathologic changes (*2*). Paraffin-embedded material from 11 of these patients was also subjected to molecular DNA analysis to demonstrate the presence of *B. anthracis* plasmid markers (*3*). These tissue samples appeared to contain 3–4 allelic variants of *B. anthracis*, based on a single variable-number tandem repeat (VNTR) marker (*vrrA*), which suggests that the material contained multiple strains of this species (*4*).

An early global comparison of the entire *pagA* gene sequence from 26 diverse *B. anthracis* isolates and a single nucleotide polymorphism (SNP)–rich region (307 bp) of the samples from Sverdlovsk found 7 SNPs in this gene (*5*). Two of these SNPs were unique to 3 of the Sverdlovsk samples. However, the distribution of the remaining 5 SNPs separated the 26 diverse isolates and the remaining Sverdlovsk samples into clusters that were consistent with diversity groups previously described by amplified fragment–length polymorphism (AFLP) analysis of a larger subset of isolates (*6*). The AFLP analysis had separated a collection of 78 isolates into 5 diversity groups. Although only 307 bp of the Sverdlovsk tissue samples were sequenced, a single SNP *(pagA* SNP 3602) placed 7 of 10 Sverdlovsk tissue samples into a large diversity group called western North America (WNA) (*5*).

Recent comparisons among 5 *B. anthracis* whole genome sequences uncovered ≈3,000 SNPs, and the rigorous examination of ≈1,000 of these SNPs across 27 diverse isolates demonstrated an extremely conserved clonal population structure for this species (*7*,*8*). These results led to a genotyping method that uses a small number of canonical SNPs (canSNPs) to replace the analysis of ≈1,000 SNPs and still precisely defines key positions and branch points within the *B. anthracis* tree (*9*). This canSNP model was recently tested against a large, diverse *B. anthracis* collection of 1,033 isolates (*10*). When all 1,033 isolates were evaluated against a panel of 13 canSNPs, the results demonstrated that each *B. anthracis* isolate had 1 of only 12 different canSNP profiles. These analyses supported the notion that a single canSNP can be used to represent an entire genome when the genome being examined is as conserved as that of *B. anthracis* (*7*–*9*). This strategy was applied to the analysis of a Sverdlovsk sample that appears to represent a single diversity group previously recognized as WNA from *pagA* sequence analysis.

## The Study

DNA was extracted by using QiaAmp DNA Mini Kits (QIAGEN, Valencia, CA, USA), following protocols recommended for formalin-fixed tissue samples. A Genomeplex Whole Genome Amplication Kit (Sigma, St. Louis, MO, USA) was used to preamplify the extracted DNA sample. Short DNA fragments were amplified from these DNA samples by using the following primer sets: canSNP.A.Br.008 F: 5′-GCCAAGATATTCGTGACATT-3′; canSNP.A.Br.008 R: 5′-TTTGGACCAGGTTTCTGTATTT-3′; canSNP A.Br.009 F: 5′-TGCGGAATATCGTTAAGTAAT-3′; canSNP A.Br.009R: 5′-TGGACGTGAATTAGGAAAAGT-3′ (for traditional PCR and sequencing); canSNP A.Br.008 F: 5′-TCTAAGAAAGATTCGCAACTACGCTATAC-3′; canSNP A.Br.008 R: 5′-TGCATTCGCAACTACGCTATACGTTTTAGATG-3′; canSNP A.Br.009 F: 5′-TGCCGGGGTTTCTACTGTGTATGTTGT-3′; canSNP A.Br.009 R: 5′-TGGGTTAGGTATATTAACTGCGGATGATGC-3′ (for mass spectrometry base composition analysis); *pagA* 981 F: 5′-AATGAGGATCAATCCACACAG-3′; and *pagA* 981 R: 5′-ATTTAAACCCATTGTTTCAGC-3′. Traditional PCR amplification schemes and DNA sequencing techniques were used for sequencing with an ABI 3100 DNA Analyzer (Applied Biosystems, Foster City, CA, USA). The mass spectrometry resequencing technique is described in detail elsewhere (*11*). Primers for real-time PCR were as follows: PagSNP.981F 5′-CACAGAATACTGATAGTCAAACGAGAACA-3′; PagSNP.981R 5′-GCACTTCTGCATTTCCATGTACTT-3′; probe 1: PagSNP.981T VIC 5′-ACAAGTAGGACtCATAC-3′; and probe 2: PagSNP.981A 6FAM 5′-CAAGTAGGACaCATACT-3′.

The Figure illustrates the relative positions of 3 canSNPs along the branches of the canSNP-derived phylogenetic tree for the 1,033 *B. anthracis* isolates (*10*). The focus of this diagram is the WNA lineage (A.Br.WNA), which forms a branch and a node part way along this branch. CanSNP typing both by traditional sequencing and by the mass spectrometry resequencing technique of the 2 positions (*10*) that define the WNA lineage (A.Br.WNA) and a branch point designated A.Br.008/009 (canSNPs in red; A.Br.008, and A.Br.009, respectively; Figure) confirms that the Sverdlovsk sample 7.RA93.15.15 (Table) is related to the WNA lineage. However, the canSNP genotype of the Sverdlovsk sample indicates that although it may share a common ancestor with the WNA lineage, it is a member of the A Branch.008/009 subgroup that comprises isolates (n = 154) that were recovered primarily from Europe and portions of Asia (*10*). The close relationship between the WNA sublineage and this major European/Asian subgroup is consistent with the hypothesis that *B. anthracis* was introduced into the North American continent by European settlers, possibly from France or Spain (*10*,*12*).

**Figure:**
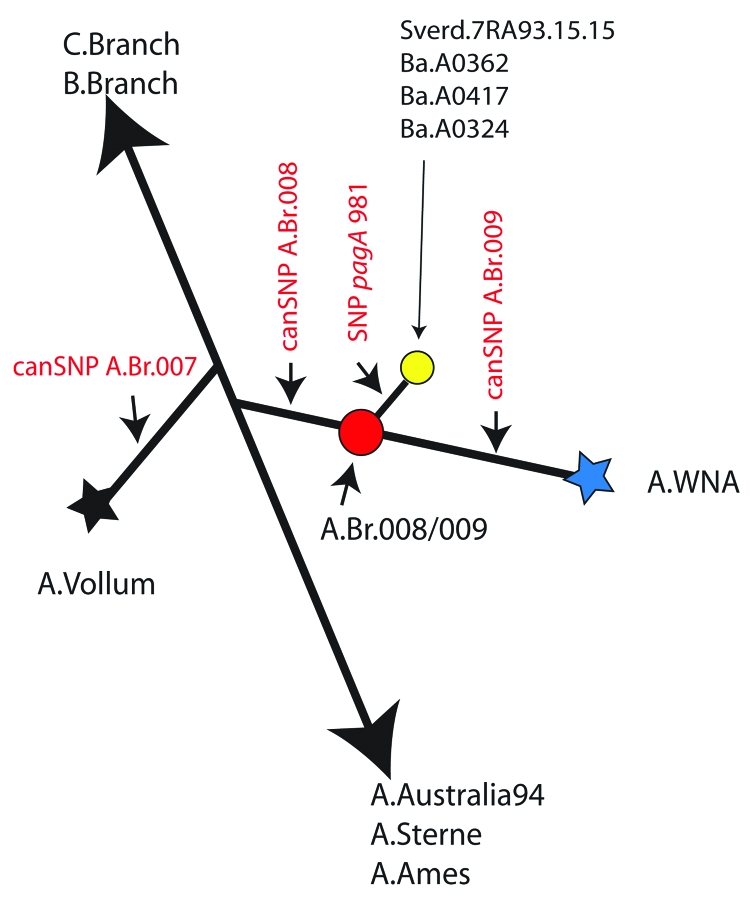
Schematic single nucleotide polymorphism (SNP) tree for *Bacillus anthracis.* This tree illustrates the relative positions of several sequenced strains of *B. anthracis* that form the specific sublineages in the A group of *B. anthracis* and in particular the Western North American lineage (A.BR.WNA, represented by a blue star [*10*]). The canonical SNPs and their positions are depicted in red lettering. A branch point (red circle) or node designated A.Br.008/009 originally represented 154 isolates and canSNP analysis places Sverdlovsk 7.RA93.15.15 in this node. The new *pagA* SNP981 defines a new branch radiating from this node and contains at least 3 other isolates.

**Table:** Canonical single nucleotide polymorphism (canSNP) typing and a new *pagA* SNP from Sverdlovsk 7.RA93.15.15 (spleen)* View Actual Table

Isolate	Country of origin	Lineage/group (*10*)	canSNP, A.Br.007	canSNP, A.Br.008	canSNP, A.Br.009	SNP, *pagA* 981
A1055		C.Br.A055	T	T	A	–pXO1
KrugerB		B.Br.KrugerB	T	T	A	A
CNEVA.9066		B.Br.CNEVA	T	T	A	A
Ames		A.Br.Ames	T	T	A	A
Sterne		A.Br.Ames	T	T	A	A
Australia94		A.Br.Aust94	T	T	A	A
Vollum		A.Br.Vollum	C	T	A	A
(Branch point)		A.Br.008/009	T	G	A	
Sv7,RA93.15.15 (spleen)		A.Br.008/009	T	G	A	T
Sv31.RA93.39.3						T
Sv40.RA93.40.5						T
Sv25.RA93.031						T
Sv1.RA93.42.1						T
Sv33.RA93.20.5						T
Sv21.RA93.38.4						T
Western North America		A.Br.WNA	T	G	G	A
A0362	Norway	A.Br.008/009	T	G	A	T
A0417	Hungary	A.Br.008/009	T	G	A	T
A0324	Slovakia	A.Br.008/009	T	G	A	T
A0293	Italy	A.Br.008/009	T	G	A	A
A0463	Pakistan	A.Br.008/009	T	G	A	A
A0149	Turkey	A.Br.008/009	T	G	A	A
A0264	Turkey	A.Br.008/009	T	G	A	A
A0032	China	A.Br.008/009	T	G	A	A
A0033	China	A.Br.008/009	T	G	A	A
A0241	Turkey	A.Br.008/009	T	G	A	A
A0245	Turkey	A.Br.008/009	T	G	A	A
A0463	Pakistan	A.Br.008/009	T	G	A	A

*This table illustrates 2 canSNPs that distinguish the subgroup/branch point A.Br.008/009 from the Western North American (WNA) sublineage. The table also depicts the status of the pagA 981 SNP in 7 of 12 Sverdlovsk samples and several other key isolates. Black shading, WNA lineage; gray shading, subgroup A.Br.008/009.

The early *vrrA* and *pagA* gene studies on Sverdlovsk tissue samples suggested that they contained multiple strains of *B. anthracis* because several *vrrA* alleles and *pagA* genotypes were noted. However, a dominant *vrrA* allele (4 repeats), as measured by both the quantity and the quality of the PCR products, was found in 9 of 13 samples from the tissue specimens (*3*). In some samples, it was the only allele present. Similarly, the *pagA* diversity group V signal (*pagA* SNP 5 at bp 3602 or bp 1791 from the ATG start) for the WNA subgroup appeared in 7 of 10 samples of the Sverdlovsk tissues (*5*). Sverdlovsk sample 7.RA93.15.15 contained these dominant allele signals, which suggests that these signals represent a single, specific strain of this pathogen.

In addition to the analysis of the canSNPs that define the A.Br.008/009 sublineage, large portions of the *pagA* gene sequence were reconstructed from sample 7.RA93.15.15 by using 40 overlapping PCR fragments without whole genome amplification. The reconstruction of this gene as well as other loci was not always feasible for many of the remaining Sverdlovsk samples because of limited DNA. Sequence analysis of these PCR products showed a new A–T synonymous transversion mutation (Table) at *pagA* position bp 981, where the start ATG = bp 1, 2, 3, or position 2784 by using the coordinates described by Price et al. (*5*). This SNP was not found in any of the original 27 diverse *B. anthracis* isolates (*5*) or in a recent *pagA* gene sequencing analysis of 124 archival *B. anthracis* samples from the Centers for Disease Control and Prevention (*13*). The status of the new *pagA* 981 site was also analyzed in a panel of isolates containing 89 diverse multiple-locus, variable-number, tandem-repeat analysis genotypes and an additional 154 A.Br.008/009 subgroup (Eurasian) isolates by using a custom made real-time PCR assay: TaqMan Minor Groove Binding probes and primers for SNPs (Applied Biosystems) (*14*). These analyses indicate that the *pagA* 981 A–T transversion represents a rare SNP allele found in only 3 Eurasian isolates and the Sverdlovsk 7.RA93.15.15 sample. (The Table shows isolates with the rare allele and a sampling of the additional Eurasian isolates.) This real-time PCR protocol is applicable to low copy–number templates and, somewhat, to mixed sample analyses. We applied it to DNA extracted from the remaining 12 Sverdlovsk tissue samples (*3*). Seven of these templates supported extensive amplification and detected only the “T” allele at this SNP locus (Table) with no evidence for the alternate “A” allele.

The tissue samples with *pagA* 981 SNP real-time PCR amplification were the same as samples that displayed the single *vrrA* 4-repeat signal (group V), except for 1 sample (40.RA93.40.5, spleen) that contained a mixture of the *vrrA* 2, 4, and 5 repeats. With this exception, a mixed signal could not be predicted from the previous results (*3*,*5*). Why a mixed signal was not seen in the latter sample is not clear, although multiple explanations exist, including the loss or curing of plasmid in certain genomes during mass culture. The remaining samples with multiple *vrrA* alleles did not produce amplicons in the *pagA* 981 real time assay.

## Conclusions

CanSNP typing provides insight into a dominant strain involved in the 1979 Sverdlovsk anthrax outbreak and identifies it as part of a large cluster of isolates (A.Br.008/009) that are found in Europe and Asia. Our findings are consistent with claims that the weaponized strain released in Sverdlovsk (Anthrax 836) was originally isolated from a rodent in the city of Kirov, Soviet Union, in the mid-1950s (*15*). These findings are not in conflict with reports that grouped the Sverdlovsk strain with the WNA sublineage because this recently defined Eurasian cluster shares a common ancestor with the WNA isolates (*7*,*10*). Reconstruction of the *pagA* gene sequence from this Sverdlovsk sample uncovered a new SNP at position 981, which appears to be specific for a small subset of Eurasian isolates. This SNP creates a branch emanating from the A.Br.008/009 node and currently contains only 3 isolates plus the prominent Sverdlovsk genotype. The extremely conserved nature of the *B. anthracis* genome (*7*), coupled with analysis of more than 230 close and diverse isolates, suggests that the *pagA* 981 transversion represents a new canSNP that can rapidly identify the closest relatives of this distinct Sverdlovsk lineage.
